# Structural and Functional Modifications of Corneal Crystallin ALDH3A1 by UVB Light

**DOI:** 10.1371/journal.pone.0015218

**Published:** 2010-12-21

**Authors:** Tia Estey, Ying Chen, John F. Carpenter, Vasilis Vasiliou

**Affiliations:** 1 Center for Pharmaceutical Biotechnology, Department of Pharmaceutical Sciences, University of Colorado Denver, Aurora, Colorado, United States of America; 2 Molecular Toxicology & Environmental Health Sciences Program, Department of Pharmaceutical Sciences, University of Colorado Denver, Aurora, Colorado, United States of America; University of South Florida College of Medicine, United States of America

## Abstract

As one of the most abundantly expressed proteins in the mammalian corneal epithelium, aldehyde dehydrogenase 3A1 (ALDH3A1) plays critical and multifaceted roles in protecting the cornea from oxidative stress. Recent studies have demonstrated that one protective mechanism of ALDH3A1 is the direct absorption of UV-energy, which reduces damage to other corneal proteins such as glucose-6-phosphate dehydrogenase through a competition mechanism. UV-exposure, however, leads to the inactivation of ALDH3A1 in such cases. In the current study, we demonstrate that UV-light caused soluble, non-native aggregation of ALDH3A1 due to both covalent and non-covalent interactions, and that the formation of the aggregates was responsible for the loss of ALDH3A1 enzymatic activity. Spectroscopic studies revealed that as a result of aggregation, the secondary and tertiary structure of ALDH3A1 were perturbed. LysC peptide mapping using MALDI-TOF mass spectrometry shows that UV-induced damage to ALDH3A1 also includes chemical modifications to Trp, Met, and Cys residues. Surprisingly, the conserved active site Cys of ALDH3A1 does not appear to be affected by UV-exposure; this residue remained intact after exposure to UV-light that rendered the enzyme completely inactive. Collectively, our data suggest that the UV-induced inactivation of ALDH3A1 is a result of non-native aggregation and associated structural changes rather than specific damage to the active site Cys.

## Introduction

Located at the anterior surface of the eye, the mammalian cornea is an avascular tissue that serves as a protective barrier between the environment and the internal ocular structures. One of the primary sources of environmental stress for the cornea is solar radiation, specifically in the ultraviolet (UV)-range. The combination of routine exposure of the cornea to UV-radiation and molecular oxygen may cause excessive production of reactive oxygen species (ROS), leading to substantial oxidative stress and subsequent tissue damage [1]. To withstand this challenge, the cornea has developed a variety of antioxidants and repair systems to minimize the deleterious effects of ROS [2]. It is becoming increasingly recognized that aldehyde dehydrogenase 3A1 (ALDH3A1) contributes to the corneal defenses by playing critical and multifunctional roles in the protection of the cornea, and likely the entire eye, against UV-induced oxidative damage [3]. As a member of the ALDH superfamily, corneal ALDH3A1 functions to detoxify a wide range of endogenous and exogenous aldehydes including the by-products of lipid peroxidation. Because the steady state ALDH3A1 concentration in the cornea exceeds that needed simply for metabolism, additional protective roles have been proposed and/or identified for the enzyme: (i) the direct absorption of UV-light, (ii) antioxidant function either directly through the scavenging of free radicals or indirectly through the production of NADPH, (iii) maintaining corneal refractive and transparent properties as a corneal crystalline, and (iv) chaperone-like activity.

The potential protective role of ALDH3A1 via the direct absorption of UVR is of great interest. It was proposed many years ago that bovine corneal protein 54 (BCP54), then identified as ALDH3A1 [4], serves an important UV absorptive role in the cornea [5]. This proposal was later substantiated by the finding that the water-soluble protein fraction of bovine corneas accounts for only 17% of the total protein but for nearly 50% of the total absorption of UVB-light (290–300 nm), leading to the suggestion that the water-soluble proteins in the cornea be termed *absorbins* [6]. Recent studies in our laboratory with *Aldh3a1* null mice clearly demonstrate that the lack of ALDH3A1 expression in the cornea results in opacification of the lens [7]. Furthermore, the UV absorbing capacity of ALDH3A1 may be intensified when bound to NAD(P)H [8], which also displays strong absorption in the near UV range. It is therefore reasonable to speculate that ALDH3A1 largely contributes to the UV-absorption properties of the cornea.

Experimental evidence supports the hypothesis that direct absorption of UV light by ALDH3A1 protects other corneal proteins by the virtue of its abundance in that tissue. In C57BL/6J inbred mice, UV-light (302 nm) caused a reduction in ALDH3A1 activity by 85% whereas other metabolic enzyme activities remained intact [9]. Similarly, a large excess of ALDH3A1 *in vitro* reduces the UV-induced inactivation of glucose-6-phosphate dehydrogenase [10]. That UV absorption by ALDH3A1 may be a major protective mechanism in the cornea is paradoxical since it is sensitive to UV-induced damage. The inactivation of ALDH3A1 by UVR to preserve other corneal elements has been termed a “suicide response” [3], [11]. UVR-induced dose-dependent inactivation of ALDH3A1 occurred in human corneal epithelial cells stably-transfected with ALDH3A1 cDNA, and purified recombinant ALDH3A1 was inactivated and covalently cross-linked by direct UV-exposure [12].

It is therefore the goal of the current research to characterize the effects of UVB-light on the biochemical and biophysical properties of purified ALDH3A1 *in vitro.* Functional studies were performed to determine how UVB-light disrupts the catalytic activity of the protein. Physical changes were assessed by quantifying and characterizing soluble aggregates with SE-HPLC and SDS-PAGE. Structural studies were performed with a variety of optical spectroscopic techniques including far UV CD, second derivative absorbance spectroscopy, and intrinsic fluorescence. Finally, UV-induced chemical modifications were detected by peptide mapping in conjunction with MALDI-TOF mass spectrometry.

## Results

### UVB-Induced Inactivation of ALDH3A1

**Figure 1:**
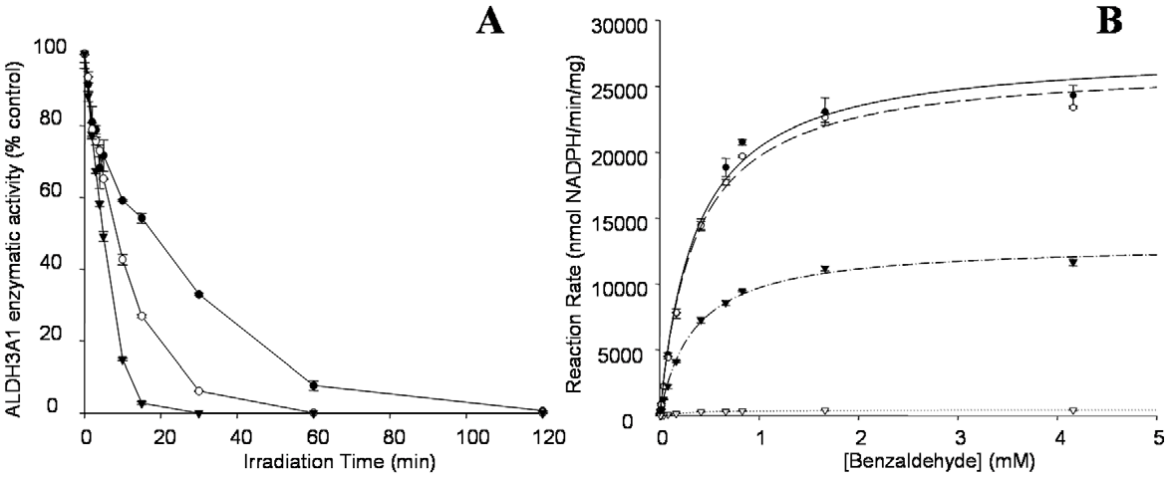
The inactivation of ALDH3A1 by 295 nm UVB-light. (***A***) The effect of protein concentration on UV-inactivation kinetics: 1 mg/mL (•), 0.1 mg/mL (○), and 0.01 mg/mL (▾). (***B***) Michaelis-Menten kinetics of UVB-exposed ALDH3A1. ALDH3A1 was irradiated at 1 mg/ml for 0 min (•), 5 min (○), 30 min (▾), and 120 min (Δ). Kinetic analysis was performed using benzaldehyde (substrate) and NADP^+^ (co-factor) in all cases. Values represent mean ± S.E. (n = 3) the error bars may be smaller than the data symbol.

During exposure to UVB-light, ALDH3A1 was inactivated in a time dependent manner. The rate of inactivation was inversely proportional to the protein concentration during the irradiation (Figure 1A). ALDH3A1 irradiated for 15 min at 1 mg/ml displayed a loss of approximately 50% of its specific activity but irradiation for the same time at 0.01 mg/ml caused a greater than 90% loss. To more fully characterize the effects of UVB-exposure on ALDH3A1 catalytic function, steady state kinetic experiments were performed with native and irradiated protein. The results for NADP^+^-dependent oxidation of benzaldehyde by ALDH3A1 are shown in Figure 1B. The native enzyme and UVB-exposed ALDH3A1 followed Michaelis-Menten saturation kinetics during the metabolism of benzaldehyde. Non-linear regression was used to fit each data set to the Michaelis-Menten model and derive the kinetic parameters *K_m_* and *V_max_* (Table 1). Native ALDH3A1 metabolizes benzaldehyde with an apparent *K_m_* of 361.9±26.7 µM and a *V_max_* of 27,754±625.8 nmol NADPH/min per mg protein. We found that UVB exposure led to a progressive reduction of the *V_max_* with time, similar to that observed with ALDH3A1 specific activity (Figure 1B). In contrast, the apparent *K_m_* remained virtually unchanged until 120 min of UVB exposure. These results suggest that UVB exposure completely inactivates the affected protein molecules, and thus reducing the population of active species.

**Table 1:** Michaelis-Menten kinetis for native and UVB-exposed ALDH3A1.

Exposure Time (min)	*V_max_* (nmol NADPH/min*mg)	*K_m_* (apparent, µM)
0	27,754±625.8	362±27
5	26,678±425.3	356±19
30	13,066±184†	347±16
120	477±11†	277±23†

Michaelis-Menten kinetic constants for UVB-exposed ALDH3A1. Data were fit to the Michaelis-Menten saturation model using the SigmaPlot® enzyme kinetics software package. Values are mean ± SE (n = 3).

† : *p*<0.05, when compared with exposure time  =  0 min.

### UVB-Induced Aggregation of ALDH3A1

**Figure 2:**
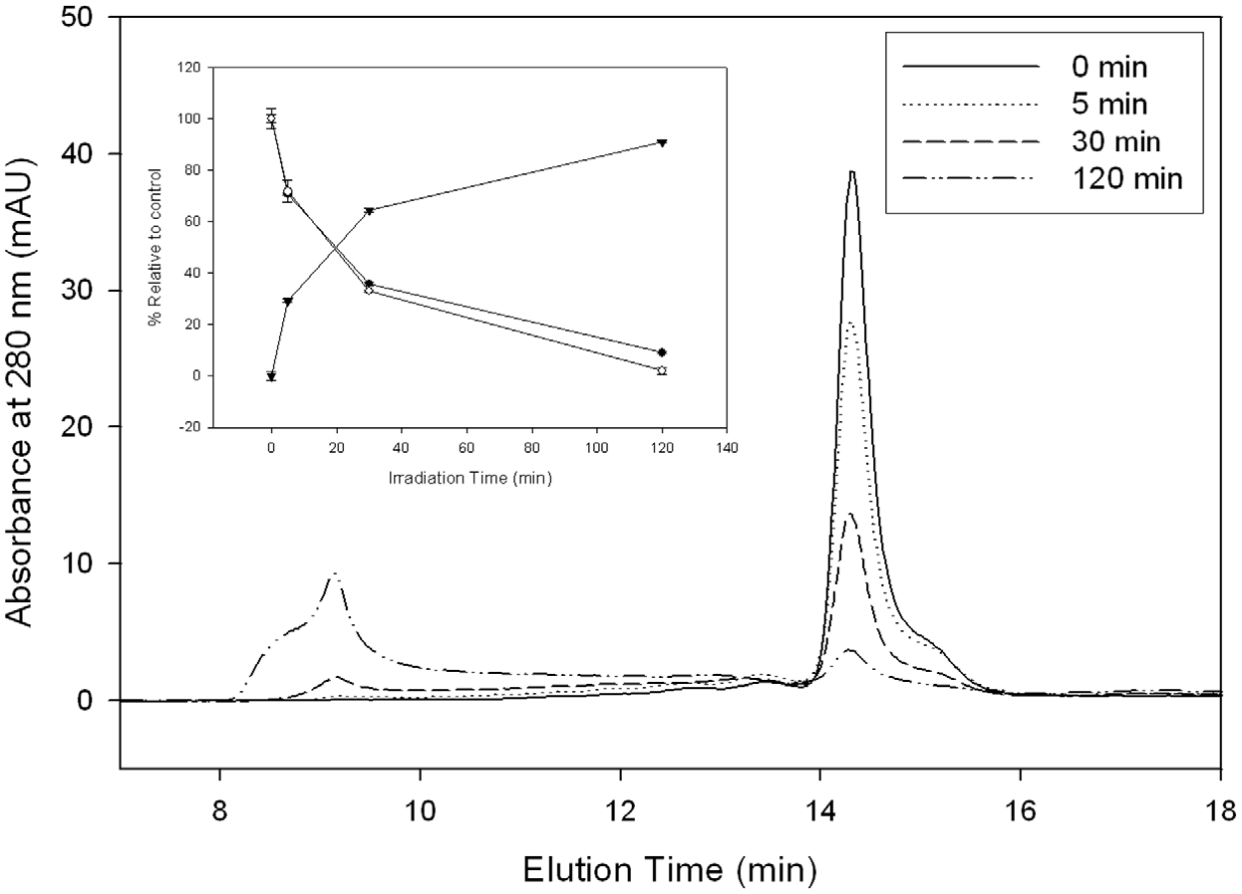
SE-HPLC analysis of UVB-exposed ALDH3A1. ALDH3A1 was irradiated for up to 120 min and then analyzed by SE-HPLC. Representative chromatograms are shown for each exposure time. *Inset:* Relationship between % ALDH3A1 dimer (by SEC, •), % ALDH3A1 soluble aggregates (by SEC, ▾), and % activity (by enzymatic assay with benzaldehyde, ○). Values represent mean ± S.E. (n = 3).

Loss of ALDH3A1 activity due to UVB exposure is most likely due to the aggregation of the protein. UVB-induced aggregation of ALDH3A1 was characterized by SE-HPLC and SDS-PAGE. Catalytically active ALDH3A1 exists as a dimer, which was found to elute at approximately 14.1 min during SE-HPLC (Figure 2). Examination of ALDH3A1 irradiated with UVB-light clearly showed the formation of large, soluble aggregates that eluted in the column void volume, suggesting a molecular weight greater than 500 kDa. Insoluble material did not pellet during preparative centrifugation (data not shown), indicating that UVB-exposure did not lead to insoluble protein aggregates. A time-dependent loss in native ALDH3A1 dimer was observed as a function of the UVB irradiation time. This loss was quantitated by integrating the dimer peak area in the SE-HPLC chromatograms and then compared to the loss of ALDH3A1 enzymatic activity (Figure 2, inset). The amount of dimer lost to soluble aggregates matched well with the loss in enzymatic activity. These results strongly suggested that activity loss of ALDH3A1 was essentially attributed to aggregate formation.

**Figure 3:**
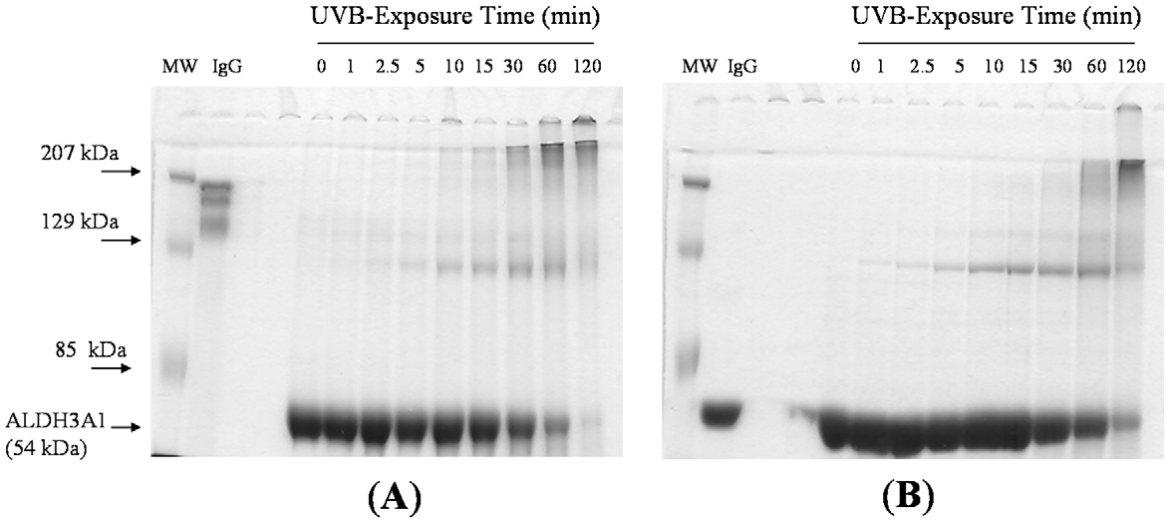
SDS-PAGE analysis of UVB-exposed ALDH3A1. ALDH3A1 was subjected to SDS-PAGE with Coomassie blue staining under non-reducing (***A***) and reducing (***B***) conditions. Each well represents 10 µg protein. Exposure times are given on the top and molecular weight markers on the left. The ALDH3A1 monomer is also indicated.

Irradiated ALDH3A1 was also subjected to SDS-PAGE analysis under both non-reducing and reducing conditions. The ALDH3A1 monomer resolved at approximately 54 kDa (Figure 3), which is in agreement with the known molecular weight of the protein. Exposure of ALDH3A1 to UVB-light led to the formation of covalently cross-linked species that resolved at apparent dimer (∼110 kDa) and higher molecular weights (>207 kDa) under non-reducing conditions (Figure 3A), some of which did not migrate through the gel and remained trapped within the well. These new species were detectable as early as 1 min after UVB-exposure and progressively increased in amount with irradiation time. By 120 min, nearly all of the ALDH3A1 protein resolved at molecular weights higher than that of the monomer. Though a small amount of these cross-linked species were dissolved under reducing conditions (Figure 3B), the majority of the bands were still present indicating that most of the cross-links were not due to disulfide bonds. This conclusion was verified by subjecting the same samples to more aggressive reducing conditions, 2.86 M β-ME and 10 min boiling or 200 mM dithiolthreitol and 10 min boiling, after which the bands for higher molecular weight species were still present and with the same intensity (data not shown).

### UVB-Induced Conformational Changes of ALDH3A1

**Figure 4:**
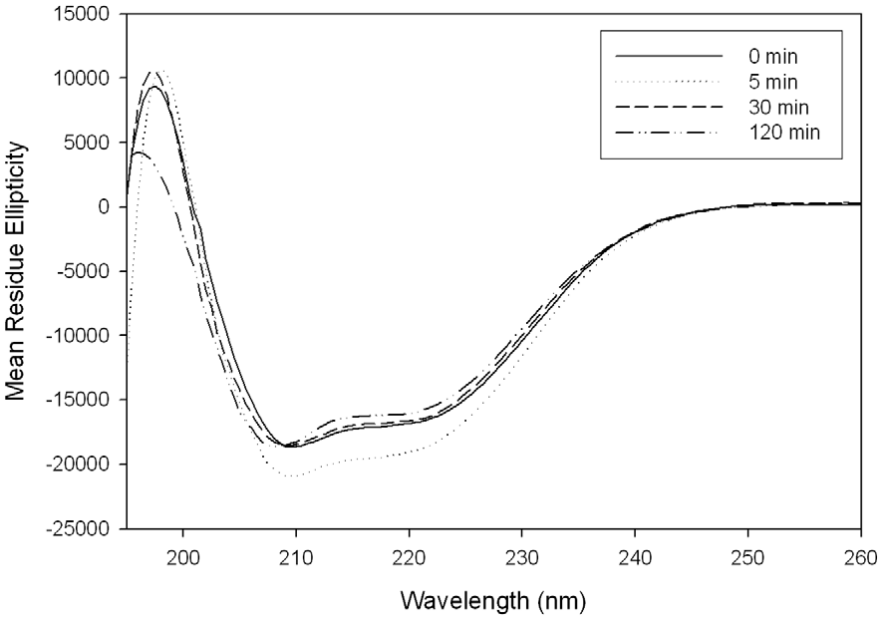
Far UV CD of ALDH3A1. ALDH3A1 (0.1 mg/ml) scans were collected from 190 to 260 nm at 0.5 nm intervals. Data transformed to mean residue ellipticity. Each spectrum is an average of three independent samples.

To gain insight into the effects of UV-induced protein aggregation on the conformation of ALDH3A1, the consequences of UVB-exposure on the structure of ALDH3A1 was characterized using several optical spectroscopic techniques. First, the secondary structure of ALDH3A1 was accessed by far UV CD. The crystal structure of ALDH3A1 from Rattus norvegicus (Protein Data Bank 1AD3) has been solved and the secondary structure has been categorized as an α/β protein with 38.9% α-helix and 15.5% β-sheet [13]. Based on the high homology between the human and rat amino acid sequences [14], it is expected that human ALDH3A1 would contain comparable secondary structures. The far UV CD of the recombinant human ALDH3A1 demonstrates two strong negative bands at 208 and 222 nm as well as a strong positive band near 195 nm (Figure 4). These spectral properties are characteristic of a protein with a significant amount of both α-helix and β-sheet structures [15]. Deconvolution of the far UV CD data using CDPro software confirms the visual inspection of the spectra with the following structural estimates: 43.8% α-helix, 4.2% β-sheet, 28.2% turn, and 23.8% random.

Far UV CD was then used to compare the structure of native and UVB-exposed ALDH3A1. In the initial UVB exposure times (up to 5 min), a notable increase in the intensity of the bands at 208 and 222 nm (associated with α-helix) was seen (Figure 4). This can be attributed to an increase in protein-protein association, as in the case of protein aggregation, which strengthens the electronic transition in the protein backbone and results in an increase in signal [16]. At longer exposure times (30 min to 120 min), both the 208 and 222 nm bands lost intensity and a substantial blue-shift in the positive band at 195 nm was observed. It should be noted that after 120 minutes of exposure to UVB, more than 90% of the protein molecules are in soluble aggregates. Therefore, the spectrum for this sample predominantly reflects the structure of protein molecules in the aggregates. These spectral changes suggest that ALDH3A1 undergoes partial unfolding due to the light-induced aggregation, and thus loses native secondary structures as α-helix and β-sheet elements are converted to random coil and/or turn.

**Figure 5:**
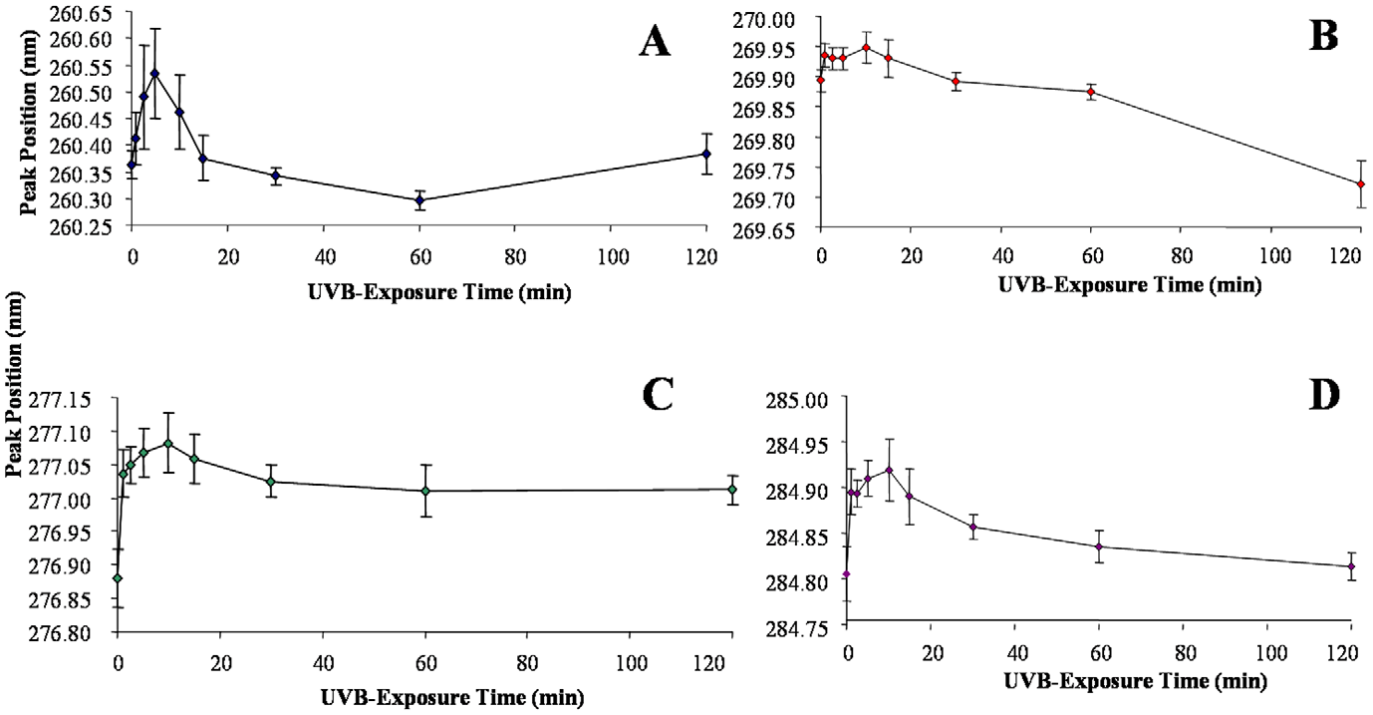
ALDH3A1 2DUV peak positions as a function of irradiation time. Peak positions representing Phe (***A***), Phe/Tyr (***B***), Tyr (***C***), and Tyr/Trp (***D***) are shown above. Values represent mean ± S.E. (n = 3) and in some cases error bars may be smaller than the data symbol.

**Figure 6:**
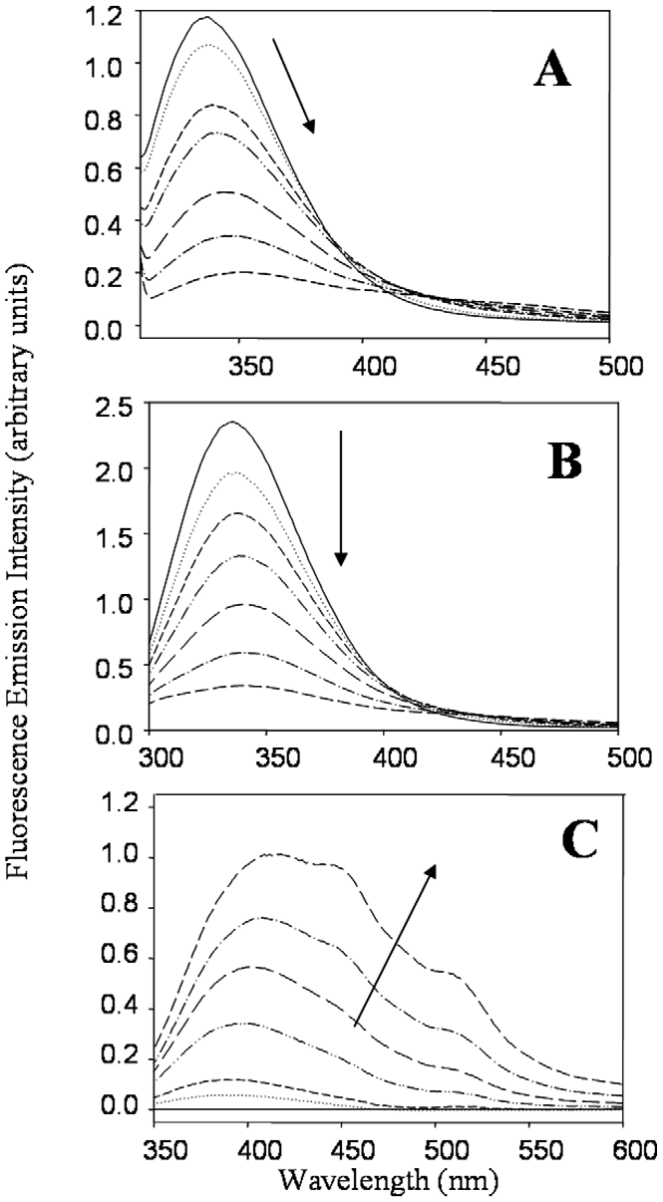
Fluoresence studies of UV-irradiated ALDH3A1. (***A***) Intrinsic ALDH3A1 Trp fluorescence (λ_ex_ = 295 nm). (***B***) Instrinsic ALDH3A1 Tyr fluorescence (λ_ex_ = 278 nm). (***C***) Fluorescence of the Trp degradation product, NFK (λ_ex_ = 315 nm). ALDH3A1 was irradiated and NFK fluorescence spectra collected at 1 mg/ml. Samples were excited at 315 nm and emission scans collected from 330 to 600 nm. The arrow indicates the trend in the change in fluorescent properties as a function of the exposure time in each case.

2DUV spectroscopy can be used to monitor the local polarity of protein aromatic residues (Trp, Tyr, and Phe), which is valuable in assessing changes in protein tertiary structure since spectral properties are affected by changes in solvent exposure of these residues [17]. The positions of four dominant peaks in the 2DUV spectra of ALDH3A1 were followed as a function of UVB-exposure time. Peaks corresponding to the solvent exposure of Phe residues (ca. 260.4 nm, Figure 5A), an overlap of Phe/Tyr residues (ca. 269.9 nm, Figure 5B), Tyr residues (ca. 276.9 nm, Figure 5C), and an overlap of Tyr/Trp residues (ca. 284.4 nm, Figure 5D) were monitored. With all of the peaks examined, there was a general trend of a shift in peak position to a longer wavelength in the early stages of exposure (1 to 15 min). A red-shift in 2DUV peak positions represents a decrease in the polarity of the local environment of the residue, which is typically interpreted as a decrease in solvent exposure [17]. In the case of ALDH3A1, the solvent exposure of the residues decreased, or become more buried, in the initial stages of UVB-exposure. This may be the result of aggregation of the protein molecules, which cause previously-exposed residues to become buried within the aggregate. At later UVB-exposure times (20 to 120 min), the peak positions shift to shorter wavelengths. This result suggests that the protein molecules may become partially unfolded after longer periods of exposure to UVB-light and resulting aggregation. Intrinsic fluorescence also can be used to monitor protein tertiary structure. Human ALDH3A1 contains 17 Tyr and 4 Trp residues. The Trp residues were selectively excited at λ_ex_ = 295 nm. The intrinsic emission of native ALDH3A1 showed a broad peak with a maximum at 338 nm (Figure 6A). Upon UVB-exposure, the intensity of the fluorescence signal progressively decreased with a notable red-shift in emission peak position. The red-shift in the Trp emission peak position could be the result of a change in ALDH3A1 tertiary structure as such transitions are often due to an increase in solvent exposure and often related to unfolding [18]. Tyrosine fluorescence was also examined and found to decrease as a function of UVB-exposure time with no apparent shift in the maximum emission wavelength (Figure 6B).

In addition to marking changes in protein structure, a loss in fluorescence emission intensity also can be due to irreversible chemical modification of the absorbing residue. UV-induced oxidation of Trp, for example, can lead to the formation of N-formylkynurenine (NFK), a doubly oxidized Trp degradation product that has unique fluorescent properties due to disruption of the indole ring; NFK has an excitation maximum of approximately 315 nm, which is at a substantially longer wavelength than that typical of Trp [19]. Thus, we also investigated the formation of NFK by exciting UVB-exposed ALDH3A1 samples at 315 nm. As anticipated, native ALDH3A1 did not display any significant fluorescence under the conditions for NFK excitation but UV-exposed protein show this fluorescent property after 2.5 min of exposure (Figure 6C). Our findings suggest that the loss of native ALDH3A1 Trp fluorescence may at least be partially explained by the degradation of the affected Trp residues into NFK.

**Figure 7:**
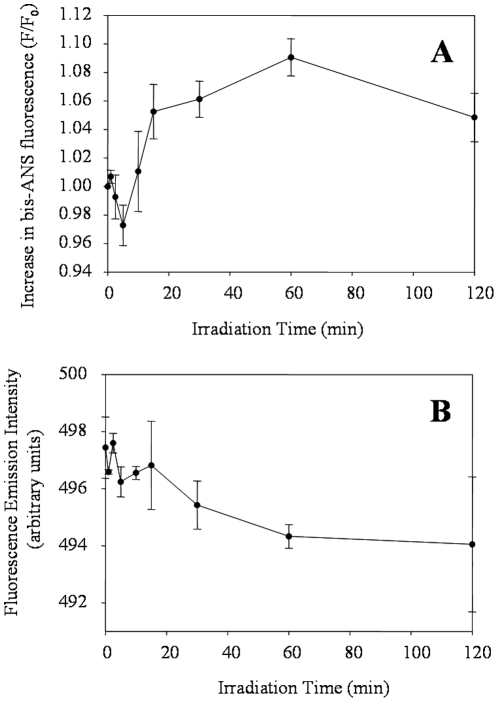
Fluorescence of bis-ANS in the presence of ALDH3A1. Native and irradiated ALDH3A1 protein was incubated with 45 µM bis-ANS, and samples were excited at 375 nm. (***A***) The relative change in bis-ANS fluorescence intensity and (***B***) bis-ANS emission peak maximum as a function of UVB irradiation time are shown. Values represent mean ± S.E. (n = 3).

Changes in the surface hydrophobicity of ALDH3A1 were monitored using the extrinsic fluorescent probe bis-ANS. Folding intermediates and molten globule states have been detected and characterized by employing bis-ANS due to the preference of the probe to bind to hydrophobic pockets on the surface of protein molecules [20]. Such pockets are likely to exist on the surface of many proteins and can increase upon the loss of native structure, which translates into an increase in bis-ANS signal intensity in the early stages of unfolding. Studies with bis-ANS and UVB-exposed ALDH3A1 revealed a decrease of bis-ANS fluorescence in the early stages of exposure (up to 5 min) when compared to the initial degree of bis-ANS fluorescence (Figure 7A). Bis-ANS fluorescence then increased slightly after 15 min of exposure and then leveled off after 30 min exposure. In addition, a shift in the emission peak maximum of bis-ANS was observed from approximately 497.5 nm (control) to 491.3 nm (120 min UVB-light) (Figure 7B). The initial decrease in bis-ANS fluorescence suggests that the accessibility of hydrophobic patches on the surfaces of ALDH3A1 was reduced during the early stages of UVB-exposure. It is possible that this result could be the consequence of the observed UV-induced protein aggregation, which would sequester hydrophobic surfaces within protein-protein interfaces and thus prevent interaction with bis-ANS. In contrast, the increased fluorescence of bis-ANS at later exposure times is likely due to an increase in hydrophobic patches on the surface of ALDH3A1 as UVB-light induces the partial unfolding of the protein structure. This conclusion is consistent with the results from far UV CD (Figure 4) as well as 2DUV (Figure 5) spectroscopic analyses, and suggest that after aggregation protein molecules may undergo additional structural rearrangement during UVB exposure.

### UVB-induced chemical modifications to ALDH3A1

**Figure 8:**
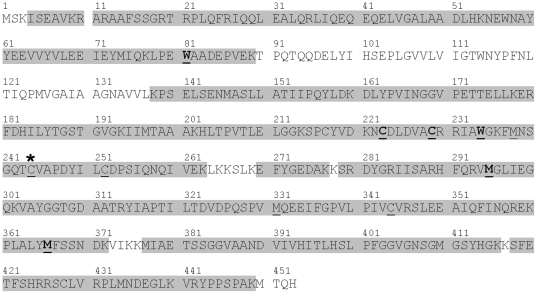
ALDH3A1 sequence coverage by LysC peptide mapping. Residues that are covered in the peptide map are shown in gray. Residues that degraded under forced oxidation (*underline*) and from UVB-light (*bold*) are shown. *: Active site Cys (Cys244).

To further characterize the UV-induced chemical modifications to ALDH3A1, peptide mapping with MALDI-TOF mass spectrometry was utilized. A peptide map of ALDH3A1 was first developed using LysC, a commonly used digestion enzyme that specifically cleaves the carboxylic side of Lys residues in peptides and proteins. The theoretical LysC digestion peptides of ALDH3A1 are shown in Table 2 and include the theoretical mass-to-charge ratio (m/z), the cleavage position, and the primary sequence of each of the anticipated peptides. Optimizing the experimental parameters of the digestion, a 1:25 digestion ratio (LysC:ALDH3A1) in 1 M KHPO_4_/1.2 M GdnHCl (pH 7.5) for 48 hr at room temperature resulted in approximately 64% sequence coverage based on the theoretical primary sequence of the protein. We were able to substantially increase the sequence coverage of the LysC peptide map by employing a fractionation method to elute the digestion peptides from the ZipTip and by collecting mass spectra in both linear and refractor modes [21]. This approach increased the sequence coverage of ALDH3A1 to over 85% (Table 3) and captured the majority of the residues of interest for chemical modifications, including three of the four Trp residues in addition to the active site Cys residue (Figure 8).

**Table 2:** Theoretical LysC peptide map of ALDH3A1.

Peptide ID	Theoretical *m/z*	Position	Primary Sequence
	[M+H]^+^		
1	6345.26	303–360	VAYGGTGDAATRYIAPTILTDVDPQSPVMQEEIFGPVLPIVCVRSLEEAIQFINQREK
2	5305.80	90–138	TPQTQQDELYIHSEPLGVVLVIGTWNYPFNLTIQPMVGAIAAGNAVVLK
3	5142.82	10–54	RARAAFSSGRTRPLQFRIQQLEALQRLIQEQEQELVGALAADLHK
4	3967.94	377–416	MIAETSSGGVAANDVIVHITLHSLPFGGVGNSGMGSYHGK
5	3013.41	237–263	FMNSGQTCVAPDYILCDPSIQNQIVEK
6	2954.38	55–77	NEWNAYYEEVVYVLEEIEYMIQK
7	2817.51	279–302	SRDYGRIISARHFQRVMGLIEGQK
8	2722.36	418–440	SFETFSHRRSCLVRPLMNDEGLK
9	2433.26	139–160	PSELSENMASLLATIIPQYLDK
10	1958.05	161–178	DLYPVINGGVPETTELLK
11	1779.90	179–194	ERFDHILYTGSTGVGK
12	1719.84	222–236	NCDLDVACRRIAWGK
13	1385.68	361–372	PLALYMFSSNDK
14	1383.68	78–89	LPEWAADEPVEK
15	1264.73	203–214	HLTPVTLELGGK
16	1014.57	441–449	VRYPPSPAK
17	958.42	270–277	EFYGEDAK
18	818.48	195–202	IIMTAAAK
19	811.37	215–221	SPCYVDK
20	646.38	4–9	ISEAVK

Theoretical LysC digestion peptides of ALDH3A1. The digestion is based upon the primary sequence of ALDH3A1 as defined by Swiss-Prot accession P30838. The peptide ID, the theoretical *m/z*, the location of the LysC cleave site, and the primary sequence is shown for each peptide. Note that the monoisotopic singly charged species, described as [M+H]^+^, is assumed for each of the digestion peptides due to the nature of ionization during MALDI-TOF analysis. Peptides > 600 *m/z* are not shown.

**Table 3:** Sequence Coverage of ALDH3A1 By Fractionation Elution^a^.

		Observed *m/z* b during fractionation elution				
Peptide ID	Theoretical *m/z* b	10% ACN	20% ACN	30% ACN	50% ACN	90% ACN
1	6345.26				*6347.01* c	*6347.24*
3	5142.82				*5144.07*	*5143.34*
4	3967.94			3970.52	3971.36	3970.27
5	3013.41		3011.53	3013.44	3013.75	3014.62
6	2954.38			2953.19	2953.87	
7	2817.51			2817.62	2817.89	2817.70
8	2722.36			2722.75	2722.68	2722.91
9	2433.26		2434.23	2435.27		
10	1958.05		1958.07	1958.02	1958.15	1958.08
11	1779.90	1779.94	1779.90	1779.92	1779.99	1779.92
12	1719.84		1717.84	1717.83	1717.87	1717.84
13	1385.68			1385.67	1385.74	1385.68
14	1383.68	1383.70	1383.69	1383.66		
15	1264.73	1264.71	1264.72	1264.73		
16	1014.57	1014.57	1014.56	1014.56		
17	958.42	958.40	958.40			
18	818.48	818.42	818.42			
19	811.37	811.32	811.34	811.31	811.37	
20	646.38	646.33				

a LysC digestion peptides were eluted from the C_18_ ZipTip by acetonitrile (ACN) fractionation as described in the methods section.

b Expressed as monoisotopic singly charged species, [M+H]^+^.

c Peptide masses that were detectable only using the linear mode of the indicated fraction are shown in italics.

**Figure 9:**
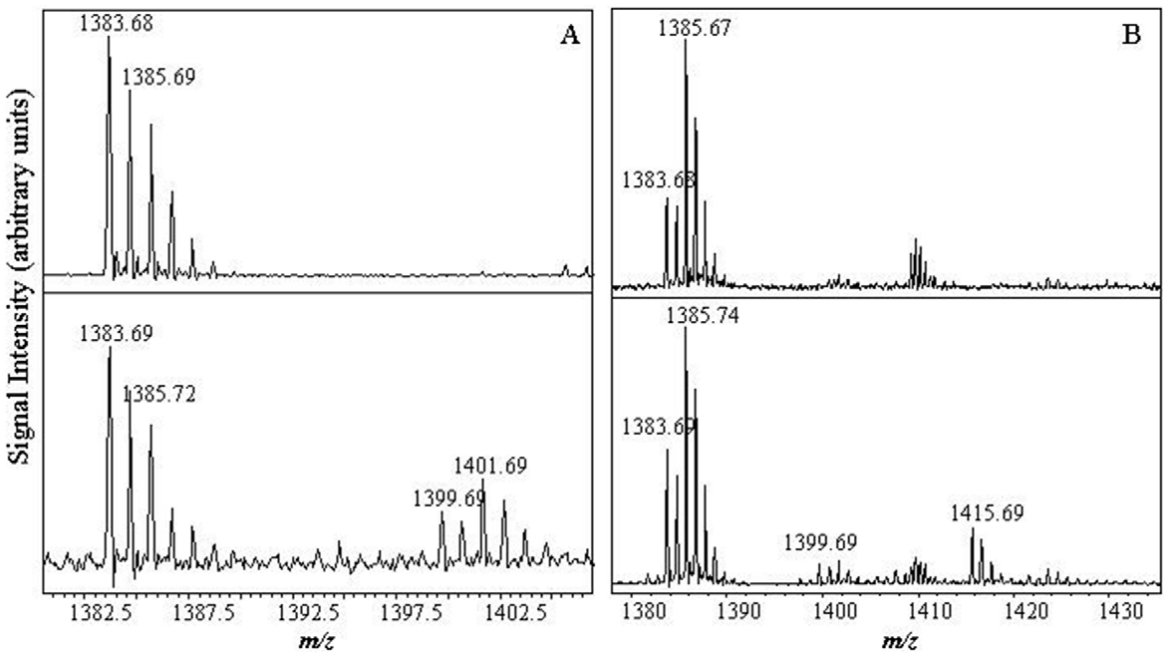
UV-induced degradation of ALDH3A1 peptides 13 and 14. After 5 min, two new masses were found representing single oxidation of each peptide (***A***). The double oxidation of peptide 13 (1415.69 *m/z*) was found after 15 min exposure (***B***). The intact *m/z* of each peptide is given on the top panel and the modified peptide(s) are shown in the bottom panel. Units have been removed from the y-axis for clarification purposes.

LysC peptide mapping was then applied to irradiated ALDH3A1 to determine which residues were modified by UVB-light. We anticipate that Trp residues would become oxidized after exposure to UVB-light because of the absorbance spectrum of Trp in the near UV region, the fluorescence studies demonstrating loss in intrinsic Trp emission (Figure 6A) and the formation of a new fluorescent entity (likely NFK, Figures 6C). Peptide 14 (1383.68 m/z) contains Trp 81 and was found to degrade after 5 min of UV-exposure as indicated by the detection of a new peptide at 1399.69 m/z. This peptide mass represents a +16 m/z mass shift compared to that for the native peptide which is due to the addition of an oxygen atom (Figure 9A). At later exposure time points (i.e. 15 min), an additional degradation product was found in the MADLI-TO mass spectrum (1415.69 m/z, Figure 9B). Compared to the intact m/z of the native ALDH3A1 peptide 14, this new peptide represents a mass shift of +32.0 m/z and suggests the double oxidation of Trp81 to NFK (Figure 9B). Thus, the changes in the properties of ALDH3A1 Trp fluorescence upon UV-exposure can be corroborated by the detection of the degradation of Trp81 to NFK in the mass spectrometry data.

**Figure 10:**
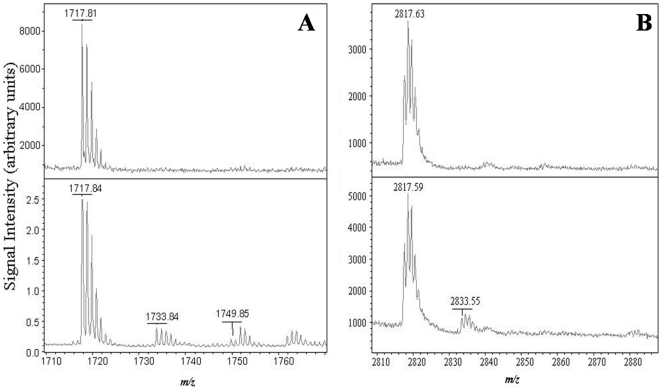
UVB-induced degradation of ALDH3A1 peptides 12 and 7. (***A***) The intact peptide 12, 1717.81 *m/z*, is shown in the top panel. After 15 min of UVB-exposure, two new peptides (1733.84 and 1749.85 *m/z*) were detected, which represent single and double oxidation, respectively (*bottom panel*). (***B***) The intact peptide 7, 2817.63 *m/z*, is shown in the top panel. A new peptide (2833.55 *m/z*) was detected after 30 min UVB-exposure due to the single oxidation of this peptide (*bottom panel*).

We also monitored the UV-induced modifications in the other Trp residues of ALDH3A1. Peptide 12 (1717.81 m/z) contains Trp234 as well as 2 Cys and was found to degrade after 15 min of UVB-exposure based on the formation of two new peptides, 1733.84 and 1749.85 m/z, which represent the single (+16.0 m/z) and double (+32.0 m/z) oxidation of the peptide, respectively (Figure 10). It is unclear from this data, however, if these modifications arise from the oxidation of the Trp or Cys residues as both are susceptible to both single and double oxidation. Trp57 (peptide 6) remained intact during exposure to both H_2_O_2_ and UV-exposure, which indicates that this residues were not oxidized under such conditions (data not shown). Finally, Trp 114 (peptide 2) could not be monitored during these studies since this peptide was not captured in the LysC peptide map (Table 3).

Methionine residues are also susceptible to UV-induced degradation and may form either singly oxidized (methionine sulphoxide, +16 m/z) or doubly oxidized (methionine sulphone, +32 m/z) species [22]. We were able to detect UV-induced damage to two ALDH3A1 LysC peptides that are suggestive of methionine oxidation. For example, Met366 (peptide 13, 1385.67 m/z) may degrade to methionine sulfoxide based on the detection of a new peptide (1401.69 m/z) in the MALDI-TOF mass spectrum after 15 min of UVB-exposure (Figure 9). Peptide 7 (2817.63 m/z) also contains a Met residue and was found to be oxidized after 30 min of UV-exposure by the formation of a new peptide with an m/z of 2833.55 (Figure 10), which represents a mass shift of +15.92 m/z arising from the single oxidation of the peptide. It is important to note, however, that both peptides 7 and 13 also contain Tyr residues. It has been documented that Tyr may also undergo photo-oxidation reactions to form Phe or related derivatives (i.e. 3,4-dihydroxy phenylalanine, DOPA) though such modifications are less common compared to Met oxidation reactions [23].

**Figure 11:**
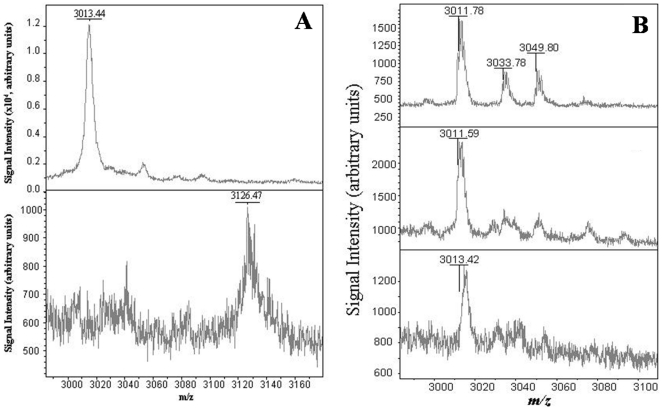
Degradation of ALDH3A1 peptide 5. (***A***) H_2_O_2_ forced degradation of peptide 5. The intact *m/z* of the native peptide is shown in the top panel and the modified peptide is shown in the bottom panel. The unit values for y-axis have been removed for clarity. (***B***) ALDH3A1 peptide 5 after UVB-exposure using MALDI-TOF. Native ALDH3A1 peptide 5 (*top panel*, 3011.78 *m/z*). This peptide was detected with no apparent mass shifts after 60 (*middle panel*) and 120 min (*bottom panel*) UVB-exposure, suggesting that the active site Cys residue is chemically intact.

In addition, we wanted to investigate the chemical integrity of the active site Cys244 residue, which is located on peptide 5 (3013.44 m/z) as the oxidation of this residue could contribute to the observed loss in ALDH3A1 activity upon UVB-exposure. Forced oxidation studies with H_2_O_2_ showed that peptide 5 is indeed susceptible to oxidation with the apparent complete conversion of the native peptide to a new peptide with a m/z of 3126.47 (Figure 11A). This shift in mass indicates the addition of seven oxygen atoms to the peptide, which can arise from various oxidation combinations to the Met, Tyr and two Cys residues of the peptide. On the contrary, after 120 min of UVB-exposure, a duration that inactivated ALDH3A1 almost completely, peptide 5 was still observed to be intact in the MALDI-TOF spectrum (Figure 11B). Re-activation experiments using 50 mM β-mercaptoethanol, which has been used to recover ALDH enzyme activity related to the oxidation of the active site Cys [24], did not result in a recovery of ALDH3A1 activity after UVB-exposure (data not shown). Collectively, these data clearly show that modification (i.e. oxidation) to the active site Cys244 residue of ALDH3A1 is not the source of the observed UVB-induced inactivation of the enzyme.

## Discussion

Protein oxidation arises from the covalent modification of a protein molecule through reactions with ROS and/or ROS by-products [23]. Under physiological conditions, oxidized proteins may function as signaling molecules in regulating cellular events, such as cell growth, differentiation, and inflammation response [25]. In other cases, oxidation of protein residues that are structurally or functionally crucial may compromise protein functions, leading to cellular damage [26]. Protein oxidation induced by UVR represents a distinct example of oxidation often referred to as photo-oxidation. Protein photo-oxidation occurs because protein molecules absorb UV energy due to electronic transitions of the peptide bond (ca. 190–220 nm) and aromatic residues Trp, Tyr, and Phe (250–300 nm). Other residues (His, Cys, and cystine) as well as bound chromophores (i.e. NADPH) can also significantly contribute to protein absorption in the near UV wavelengths. Photo-oxidation can proceed through both direct (Type I) and indirect (Type II) mechanisms [27]. Direct photo-oxidation results in photo-ionization of the absorbing species and the consequent formation of an excited species, which can then undergo electron transfer with suitable acceptors to form radicals. Inherently unstable, these radicals rapidly react with molecular oxygen to form peroxides and other degradation products. Di-tyrosine cross-linking, Trp degradation to NFK, and disulfide scrambling are some examples of the end products of direct protein photo-oxidation reactions [22], [23], [27]. Indirect photo-oxidation is characterized by the formation of singlet oxygen that arises from energy transfer from the absorbing species specifically to molecular oxygen. Though indirect oxidation leaves the absorbing species intact, singlet oxygen is free to react with most protein residues, although Trp, His, Tyr, Met, and Cys demonstrate the highest reaction rate constants [23]. It is therefore not surprising that the products of protein photo-oxidation are diverse and often protein-specific.

Our current research shows that ALDH3A1 is sensitive to inactivation by UVB-light. The mechanism of ALDH3A1 catalysis involves the hydride ion transfer from the aldehyde substrate to the nicotinamide ring of the co-factor NADP^+^, which in turn requires the activation of the active site Cys244 to a thiolate ion [28]–[30]. Thus, the enzymatic activity of ALDH3A1 depends on Cys244 being in the reduced state for activation and oxidation of Cys244 would lead to loss of metabolic capacity. UVB-induced inactivation of ALDH3A1 was not averted by incubation with the reducing agent β-ME, suggesting that the observed inactivation is not directly related to the reversible oxidation of Cys244. This was further supported by LysC peptide mapping and MALDI-TOF mass spectrometry analysis, in which Cys244 remained intact after even 120 min of UVB exposure (Figure 11B). This clearly demonstrates that the active site Cys244 maintains its chemical integrity during UVB-exposure. Rather, our results document that UV-induced loss of ALDH3A1 catalytic activity is a result of aggregation of the protein during exposure to UVB-light. The conversion of ALDH3A1 dimers to soluble aggregates correlates directly with the loss in ALDH3A1 activity (Figure 3). These UVB-induced aggregates are covalently crosslinked, although only a small fraction of the crosslinks can be attributed to intermolecular disulfide bonds. Other researchers have also observed non-reducible crosslinks upon UV-irradation of proteins [31], including the lens crystallins [32], [33], though the origin of such covalent modifications remains an area of debate. Protein photochemistry reveals that there are many possibilities as the termination reaction of protein-based radicals can lead to crosslinking [23], [32]. Di-tyrosine [32], [34], di-tryptophan [35], and Trp-Tyr crosslinks [36] may all arise from the termination reactions of radicals formed during direct oxidation. To complicate matters, singlet oxygen can induce carbonyl formation and lead to His [37], Arg [38] and Lys crosslinks [37] originating from Schiff base/Amadori product reactions [23], [38]. Though the residues involved in the UVB-induced crosslinking of ALDH3A1 were not resolved in the current study, data from the fluorescence experiments hint at the potential involvement of Trp and/or Tyr in the cross-linking. Examination of ALDH3A1 Trp fluorescence showed a progressive loss of emission spectra intensity upon UV-exposure together with the formation of a new fluorophore, which is attributed to the photo-degradation of Trp to NFK based on the results of the mass spectrometery data (see further discussion below). However, it is important to consider that both di-tyrosine [39] and di-tryptophan [35] may also exhibit similar fluorescent properties to NFK and, therefore, the changes in fluorescence due to UV-light may represent a variety of degradation products. Taken together, our results suggest that UV exposure induces chemical modifications of ALDH3A1 protein molecules, which lead to the formation of soluble aggregates and subsequent inactivation of ALDH3A1 enzyme; as such, the limiting factor for the extensiveness of UV-induced inactivation is the percent of modified ALDH3A1 molecules by the energy released from a given dose of UV light. This notion is further supported by our observation that the rate of ALDH3A1 inactivation by UV light correlates with the time of exposure and inversely with the protein concentration.

As discussed previously, Trp is a logical participant in protein photo-oxidation reactions based on the near UV absorption properties of this amino acid residue. The changes in ALDH3A1 Trp fluorescence in conjunction with the detection of a new florescent species were highly suggestive of the oxidation of Trp to NFK. Mass spectrometry was therefore utilized to detect and thoroughly characterize the effects of UVB on the four Trp residues of ALDH3A1. Our results show that Trp81 is singly and doubly oxidized to hydroxyl-Trp and NFK, respectively (Figure 9). The UV-induced degradation of Trp is complex and can lead to a variety of products and reactive intermediates while also generating ROS (specifically H_2_O_2_). These by-products can then attack other residues on the protein molecule to perpetuate the damage [23]. Though Trp may likely be responsible for initiating the UV-induced damage by the direct absorption of UV energy, it is unlikely that Trp will be the only residue to be chemically modified by photo-oxidation reactions. This is illustrated by the detection of Met259 oxidation on peptide 7 after 30 min of UVB-exposure (Figure 10B) even though Met does not significantly absorb at the irradiation wavelength of 295 nm.

The biological significance of UV-induced damage to ALDH3A1 has various implications. Our current work demonstrates that the direct absorption of light leads to the inactivation and formation of soluble aggregates of ALDH3A1. As ALDH3A1 is the primary enzyme responsible for the detoxification of aldehydes in the cornea [7] the loss of ALDH3A1 activity may severely impair the ability of this tissue to reduce and eliminate aldehydes especially under conditions of oxidative stress. The cornea, however, is at an advantage due to the abundant expression of ALDH3A1 [40], [41] combined with the broad range of substrate specificity [42], both of these factors would likely allow for aldehyde metabolisms in the event of substantial inactivation of ALDH3A1. Non-native protein aggregation can also be problematic as large oligomers can scatter light, a highly undesirable quality for a transparent tissue essential for normal vision, and also impede the removal of damaged proteins through the proteosomal degradation via direct and indirect mechanisms. Such is the case of the lens in which the accumulation of oxidized and aggregated lens proteins has been implicated in lens opacification and cataract formation [43]. Our *in vitro* UV-exposure experiments revealed that even though UV-light resulted in the formation of very large ALDH3A1 aggregates, the protein remained primarily soluble even at the extreme exposure times (120 min). These results suggest that physiologically relevant levels of UV-exposure to the cornea would not likely cause insoluble aggregation of ALDH3A1 protein and, therefore, not impact the transparent properties of the cornea.

Experimental evidence continues to support the contribution of ALDH3A1 to the defense arsenal of the cornea. Recent data from our laboratory shows that the lack of ALDH3A1 in mice does not only impact the cornea but also extends to the lens: ALDH3A1-null animals were more likely to develop corneal clouding and lens opacification when compared to age-matched control animals. In fact, the lens appeared to be considerably more sensitive to the lack of ALDH3A1 expression in these animal studies [7]. This is not entirely surprising as damage to the lens is relatively permanent due to negligible metabolic turnover in the lens fiber cells whereas the corneal epithelium is renewed approximately every two weeks. It can be logically speculated that high steady-state levels of ALDH3A1 in the cornea functions as a versatile barrier to protect the lens against UV-induced oxidative damage.

### Conclusions

ALDH3A1 is a highly expressed corneal enzyme that is susceptible to UVB-induced inactivation. UVB-exposure leads to the covalent cross-linking and aggregation of ALDH3A1 molecules and partial unfolding of the protein. These structural changes, rather than the direct modification of the active site Cys residue, are the source of UV-induced inactivation. The inactivation of ALDH3A1 by UV and other stresses may not render the cornea completely defenseless against toxic aldehydes, however, due to the abundant expression of this enzyme in the cornea as well as wide range in substrate specificity. Our data strongly suggests a novel role of ALDH3A1 as a dominant absorbing species of the mammalian cornea and plays a critical and multi-faceted role in protecting the entire eye from oxidative damage. This is collaborated with recent data showing that *Aldh3a1* knockout mice develop cataracts in the lens although ALDH3A1 protein expression is essentially confined to the cornea [7].

## Materials and Methods

### Chemicals and Materials

Potassium phosphate, Tris-HCl, potassium chloride, sodium pyrophosphate, glycerol, sodium dodecyl sulfate, Bromophenol blue, guanidine hydrochloride (Gdn HCl), EDTA, 4,4′-dianilino-1,1′-binaphthyl-5,5′-disulfonic acid dipotassium salt (bis-ANS), dithiolthreitol, NAD(P)^+^, and benzaldehyde were purchased from Sigma (St. Louis, MI). Methanol, β-ME, H_2_O_2_ and HPLC-grade acetonitrile and water (for mass spectrometry applications) were obtained from Fisher Scientific. Trifluoroacetic acid (TFA) was from Pierce (Rockford, IL). All other reagents were of analytical grade or higher and purchased from Sigma. 5′AMP Sepharose 4B column material was from Amersham Pharmacia Biotech Inc. (Piscataway, NJ). Filtration and Amicon® membranes as well as C_18_ ZipTips® were obtained from Millipore Corporation (Belford, MA). Dialysis cassettes were from Pierce Chemical Company (Rockford, IL). The HPLC column and pre-filter were purchased from Tosoh Biosciences (Montgomeryville, PA) and Rhodyne LLC (Pohnert Park, CA), respectively. Sequencing grade LysC was purchased from Wako Chemicals USA Inc (Richmond, VA). The MALDI-TOF matrix α-cyano-4-hydroxycinnamic acid was from Agilent Technologies Inc. (Palo Alto, CA).

### Expression and Purification of Recombinant Human ALDH3A1

Recombinant human ALDH3A1 was expressed in Sf9 cells using a baculovirus infection system and purified by affinity chromatography as previously described with modifications [10], [42]. Briefly, the cells were lysed by sonication and the total soluble protein fraction isolated by centrifugation and recovery of the supernatant. The soluble fraction was applied to a 5′-AMP Sepharose 4B affinity column equilibrated with binding buffer (100 mM KHPO_4_, 1 mM EDTA, 1 mM β-ME, pH 7.4), and the protein was eluted in elution buffer (binding buffer containing 0.25 mM NAD^+^). Triton X-100 was excluded from purification buffers due to its interference in mass spectrometry analysis of the protein. ALDH3A1 was concentrated using an Amicon® device (30 kDa MWCO membrane) and then dialyzed against a large volume of 20 mM Tris-HCl/100 mM KCl (pH 7.4) using a 10 kDa MWCO dialysis cassette. Protein concentration was determined by absorbance at 280 nm using the theoretical extinction coefficient (ε^0.1%^ = 1.014 ml mg^−1^cm^−1^) determined by the ALDH3A1 primary sequence (Swiss-Prot accession number P30838).

### UVB Irradiation

Purified ALDH3A1 was prepared at 1.0 mg/ml (unless otherwise noted) in 20 mM Tris-HCl/100 mM KCl (pH 7.4). Samples were placed in a 3.5 ml quartz fluorescence cuvette with 1 cm pathlength and irradiated (λex = 295 nm, 10 nm bandwidth) with gentle stirring at 25°C for 0 to 120 min in an AVIV Spectro-fluoromemeter Model ATF 105. After irradiation, samples were immediately removed from the cuvette, placed on ice and stored in the dark until analysis. Samples were analyzed within 24 hr of irradiation. As a “dark” control, protein samples were incubated under the same experimental conditions as the irradiated sample except that the light source was not turned on. Both irradiation and dark control experiments were performed in triplicate.

### Enzyme Activity Assays and Michaelis-Menten Saturation Kinetics

The catalytic activity of purified ALDH3A1 was evaluated spectrophotometrically at 25°C by the oxidation of benzaldehyde (substrate) in the presence of NADP^+^ (cofactor) as previously described [42]. The rate of NADPH production was monitored at 340 nm using a Beckman DU-640. The assay reaction mixture (1 ml) contained 0.1 M sodium pyrophosphate (pH 8.0), 1 mM NADP^+^ and 5 µg of purified protein (native or irradiated). The reaction was initiated by adding 100 µl of 50 mM benzaldehyde prepared in 10% methanol for a final concentration of 5 mM, which is greater than that needed to saturate the enzyme. The initial, linear portion of resulting rate was normalized for protein concentration and expressed as specific activity (nmol NADPH/min/mg protein). For Michaelis-Menten saturation kinetics studies, the concentration of benzyaldehyde was varied from 1 µM to 4 mM while other reaction constituents were held constant. The resulting rates were fit to the Michelis-Menten saturation model using SigmaPlot® Enzyme Kinetics software (version 7.0, 2001) to determine the apparent affinity constant (*K_m_*) and maximum velocity (*V_max_*). All enzyme assays were performed in triplicate. For ALDH3A1 reactivation experiments, ALDH3A1 was incubated with 50 mM β-ME for 30 min at room temperature in the dark based on previous reactivation studies [24]. ALDH3A1 was then immediately assayed for the recovery of specific activity with benzaldehyde as described above.

### Size Exclusion High Performance Liquid Chromatography (SE-HPLC)

A Hewlett-Packard 1090 HPLC system with a 250 µl injector syringe and a diode array UV detector was equipped with a TSK Gel-3000 SWXL column and pre-column filter. The column was equilibrated at room temperature with mobile phase (10 mM Tris-HCl/100 mM KCl, pH 7.4), which had been filtered (0.22 µ) and de-gassed prior to use. The flow rate of the mobile phase was 0.6 ml/min and protein was detected at 280 nm. Prior to analysis ALDH3A1 samples were centrifuged (10,000 *xg*). An aliquot of the supernant containing 100 µg of protein was injected onto the column. Peak area for the native protein was integrated from elution time of approximately 14 to 16 min using Chemstation^TM^ software.

### SDS-PAGE Analysis

Protein samples were analyzed by SDS-PAGE using 7.5% acrylamide gels. Protein samples (10 µg) were prepared in Laemmli sample buffer (62.5 mM Tris-HCl, pH 6.8, containing 25% glycerol, 2% SDS, and 0.01% Bromophenol blue) and boiled for 5 min (with 750 mM β-ME for reducing conditions) or 1 min (without β-ME for non-reducing conditions) prior to loading. To confirm that the reducing conditions were sufficient to disrupt disulfide bonds, 10 µg of a human IgG antibody was run as a control. Electrophoresis was conducted for 1 hr 10 min at 180 volts, and then gels were stained with Coomassie blue to visualize the protein bands.

### Far UV Circular Dichrosim (far UV CD)

Samples were prepared at a protein concentration of 0.1 mg/ml. Far UV CD spectra were collected from 190 to 260 nm at 0.5 nm intervals using a 0.1 cm path-length quartz cuvette at 25°C with an AVIV 62DS spectrometer. The spectrum for the appropriate buffer was subtracted. Raw data were transformed to mean residue ellipticity, and spectral deconvolution was performed using CD Pro software available online at http://lamar.colostate.edu/~sreeram/CDPro.

### Second Derivative UV Absorbance Spectroscopy (2DUV)

ALDH3A1 samples were centrifuged (10,000 *x*g), diluted to 0.5 mg/ml and placed in a 1 ml quartz cuvette (1 cm path-length). UV absorbance spectra were collected from 190 to 500 nm with an Agilent UV/Vis 8453 spectrophotometer using a 1 nm data spacing interval and an integration time of 25 sec. The raw absorbance data were transformed to second derivative data using Chemstation™ software using a method previously described [44].

### Intrinsic Fluorescence Studies

For intrinsic Trp fluorescence, native or irradiated ALDH3A1 was diluted to 0.05 mg/ml and protein samples were excited in a 1 cm path-length cell at 295 nm (4 nm bandwidth) using an AVIV Spectrofluorometer. Emission spectra were collected from 300 to 500 nm (8 nm bandwidth). For N-formyl-kynurenine (NFK) fluorescence, ALDH3A1 was irradiated at 1 mg/ml and samples were excited at 315 nm (4 nm bandwidth) without dilution. Emission spectra were collected from 330 to 600 nm (8 nm bandwidth). All spectra were acquired at 25°C and the appropriate buffer spectrum was subtracted.

### Bis-ANS Binding Studies

The fluorescent probe 4,4′-dianilino-1,1′-binaphthyl-5,5′-disulfonic acid (bis-ANS) has been used to characterize the surface hydrophobicity of proteins including that of partially unfolded intermediates and molten globule states [20]. ALDH3A1 was diluted to 0.1 mg/ml (approx. 2 µM) and incubated with 45 µM bis-ANS for 2 hr at room temperature in the dark. The sample was excited at 375 nm (4 nm bandwidth), and the emission spectra was collected from 380 to 650 nm (8 nm bandwidth). Buffer containing 45 µM bis-ANS only was used as the blank and its signal was subtracted from that of the protein-containing sample.

### LysC Peptide Mapping and MADLI-TOF Mass Spectrometery

ALDH3A1 (50 µg) was pipetted into 1 M KHPO_4_ (pH 7.5) containing 1.2 M GdnHCl, and 2 µg of LysC was added to the reaction for a mass ratio of 1∶25 relative to ALDH3A1. ALDH3A1 was digested for 48 hr at room temperature. Peptides were concentrated and desalted using C_18_ ZipTips® according to the fractionation elution method described by the manufacturer. Briefly, various fractions of digestion peptides were consecutively eluted from the ZipTip® by increasing the concentration of acetonitrile in the elution buffer. The elution buffers contained 10–90% acetonitrile in water with 0.1% TFA. Each fraction was mixed with an equal volume of α-cyano-4-hydroxycinnamic acid matrix containing picomole levels of calibration mass standards, spotted, and allowed to air dry. Mass spectra were collected using a Bruker MALDI-TOF mass spectrometer (Bruker Daltonics, Inc, Billerica, MA) operated in the positive ion and both linear reflector modes as indicated. The resulting peptide map was compared to the theoretical peptide map based on the primary sequence of ALDH3A1. For chemically-induced oxidation studies, ALDH3A1 was incubated with 3% H_2_O_2_ for 4 hr at room temperature in 20 mM Tris-HCl/100 mM KCl (pH 7.4). LysC peptide mapping and MALDI-TOF data collection were conducted as described above.
